# Host–Guest Chemistry for Simultaneous Imaging of Endogenous Alkali Metals and Metabolites with Mass Spectrometry

**DOI:** 10.1021/acs.analchem.1c03913

**Published:** 2022-01-25

**Authors:** Leonidas Mavroudakis, Kyle D. Duncan, Ingela Lanekoff

**Affiliations:** Department of Chemistry—BMC, Uppsala University, 751 24 Uppsala, Sweden

## Abstract

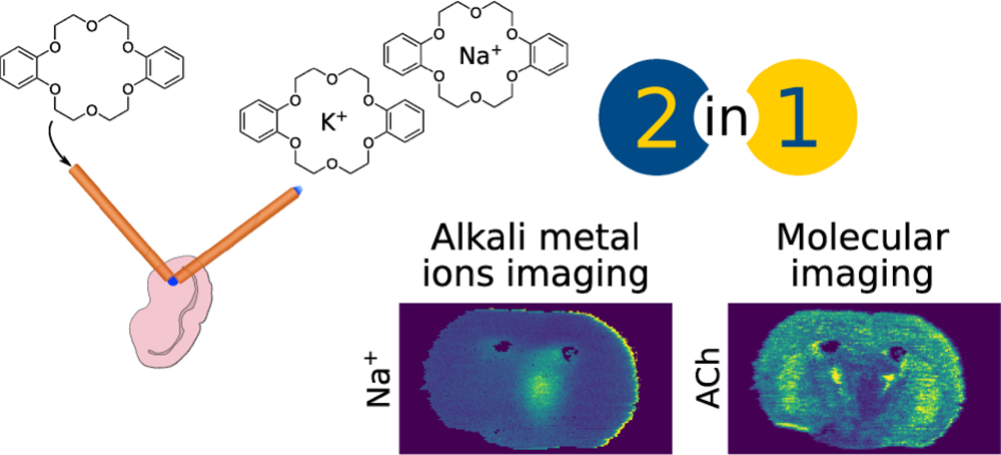

Sodium and potassium are biological alkali metal ions that are essential for the physiological processes of cells and organisms. In combination with small-molecule metabolite information, disturbances in sodium and potassium tissue distributions can provide a further understanding of the biological processes in diseases. However, methods using mass spectrometry are generally tailored toward either elemental or molecular detection, which limits simultaneous quantitative mass spectrometry imaging of alkali metal ions and molecular ions. Here, we provide a new method by including crown ether molecules in the solvent for nanospray desorption electrospray ionization mass spectrometry imaging (nano-DESI MSI) that combines host–guest chemistry targeting sodium and potassium ions and quantitative imaging of endogenous lipids and metabolites. After evaluation and optimization, the method was applied to an ischemic stroke model, which has highly dynamic tissue sodium and potassium concentrations, and we report 2 times relative increase in the detected sodium concentration in the ischemic region compared to healthy tissue. Further, in the same experiment, we showed the accumulation and depletion of lipids, neurotransmitters, and amino acids using relative quantitation with internal standards spiked in the nano-DESI solvent. Overall, we demonstrate a new method that with a simple modification in liquid extraction MSI techniques using host–guest chemistry provides the added dimension of alkali metal ion imaging to provide unique insights into biological processes.

## Introduction

Alkali metal ions such as Na^+^ and K^+^ play essential roles in biological systems for maintaining cell homeostasis. For example, in the case of ischemic damage, depletion of adenosine triphosphate (ATP) results in disruption of the Na^+^/K^+^-ATPase, and thus, intracellular Na^+^ concentration increases, while K^+^ concentration decreases.^1,2^ Hence, accurate determination of Na^+^ and K^+^ alterations is important for understanding their role in homeostasis maintenance and/or disturbance. For that purpose, atomic spectroscopy techniques, such as atomic absorption or emission, have been established for the quantitative determination of metal ions in biological samples.^3^ However, these methods require homogenization and digestion of the tissue before analysis, which leads to a loss of spatial information.

Spatially resolved information is of particular importance in biological systems to elucidate mechanisms of action involved in diseased states that lead to homeostasis perturbations.^4^ Imaging of elemental distributions can be achieved with techniques such as time-of-flight secondary ion mass spectrometry (TOF SIMS),^5^ laser ablation inductively coupled plasma mass spectrometry (LA ICP MS),^6^ X-ray fluorescence microscopy,^6^ and proton-induced X-ray emission (PIXE).^6^ For imaging of molecular distributions, several techniques have been developed, including matrix-assisted laser desorption/ionization mass spectrometry (MALDI MS),^7^ desorption electrospray ionization mass spectrometry (DESI MS),^8^ and nanospray desorption electrospray ionization mass spectrometry (nano-DESI MS).^9^ Recently, DESI followed by PIXE has been applied on the same tissue section in two experiments to obtain trace elements and molecular information on a single tissue section.^10^ The plethora of MSI and other available imaging techniques provide many opportunities;^11^ however, deeper insights with fewer experiments are gained when elemental and molecular imaging are combined simultaneously in one technique.

One reason for the limited availability of imaging techniques with simultaneous imaging of Na^+^ and K^+^ and molecular distributions is that molecular imaging in a positive ion mode requires high mass resolving power to distinguish isobars due to adduct formations. Additionally, it requires the MSI technique to eliminate matrix effects based on Na^+^ and K^+^ adducts.^12^ To circumvent the limitations, Liu et al. utilized a MALDI-TOF/TOF instrument, peaking at 40,000 mass resolution, first in the negative ion mode to image metabolites and then in the positive ion mode in a separate experiment for MSI of alkali metal ions.^13^ However, even when performing two different experiments, modern mass spectrometers with a high enough resolving power to resolve many metabolite isobars (>100,000 *m*/Δ*m* at *m*/*z* below 400), such as Orbitrap or Fourier transform ion cyclotron resonance, are not capable of scanning below 50 Da, which makes direct detection of Na^+^ and K^+^ impossible. Therefore, indirect determination of Na^+^ and K^+^ distributions through adduct formation is a reasonable approach.

Crown ethers are macroheterocyclic compounds that form high-affinity complexes with alkali metal ions. Ever since the discovery of the first crown ether,^14^ dibenzo-18-crown-6 (db18c6), the complexation properties of crown ethers with metal ions have been exploited in various fields of chemistry.^15−17^ Crown ethers have also been used with mass spectrometry for studying complexation properties and quantitation of guests.^18−20^ For example, an active pharmaceutical ingredient was complexed with crown ethers for its direct quantitative analysis in tablets by doping the solvent in DESI MS.^20^ Similarly, the extraction solvent can be doped in nano-DESI MSI with both internal standards and reagents to eliminate matrix effects^12^ from alterations in Na^+^ and K^+^ and gain quantitative, sensitive, and selective analysis.^21−29^ In nano-DESI, the extraction solvent forms a liquid bridge between two fused silica capillaries into which chemical species are desorbed upon contact with the tissue for subsequent ionization.^30^ By doping the solvent, endogenous molecules will be ionized and/or complexed simultaneously with the dopant and provide unique information even in the complex chemical matrix of thin tissue sections.

Here, we report the first use of crown ethers in MSI by adding them into the nano-DESI solvent for simultaneous elemental and metabolite imaging. We show that in situ host–guest chemistry in MSI is robust and enables quantification of Na^+^ or K^+^ in imaging of complex tissue samples. Additionally, we show that simultaneous quantitative ion images are provided for endogenous molecules by also doping the solvent with internal standards. We apply our method to study the distribution of Na^+^, K^+^, lipids, and metabolites in a middle cerebral artery occlusion (MCAO) rodent stroke model and directly quantitatively correlate their accumulation and depletion in the ischemic region.

## Materials and Methods

### Chemicals and Biological Samples

All chemicals were purchased from Sigma-Aldrich and used without further purification, unless otherwise stated. Methanol was purchased from Merck (LC-MS grade); 12-crown-4, 15-crown-5, and benzo-15-crown-5 from Fisher Scientific; and dl-glutamic-2,4,4-*d*_3_ acid from QMX Laboratories (Thaxted, UK).

Flash frozen healthy Sprague–Dawley rat brain tissue was purchased from Innovative Research Inc. (Novi, MI, USA) and sectioned at 12 μm thickness using a cryostat (Cryostar NX 70, Thermo 165 Scientific, Bremen, Germany). Flash frozen transient MCAO (1 h MCAO and 2 h reperfusion) mouse brain tissue from one 8–10 week old male C57BL/6 mouse was purchased from Creative Biolabs (NY, USA) and sectioned at 10 μm thickness (Leica CM1900, Leica Microsystems). Tissue sections were thaw-mounted on regular microscope glass slides and stored at −80 °C until the analysis of three consecutive sections.

### Preparation and Analysis of NaCl-Spiked Mimetic Tissue Model

A NaCl-spiked mimetic tissue model was prepared according to Barry et al.^31^ using rat brain homogenates spiked with differential amounts of NaCl: 0, 20, 50, and 90 μmol g^–1^. The mimetic tissue model was stored at −80 °C until cryosectioning at 14 μm thickness and thaw-mounted on regular microscope glass slides. Details on the preparation of the mimetic tissue model are provided in the Supporting Information.

A section of the NaCl-spiked mimetic tissue model was imaged using conventional nano-DESI as previously described,^23^ where 0.1 μM of dibenzo-18-crown-6 was doped in the solvent (methanol/water 9:1 v/v). The solvent flow rate was 0.5 μL min^–1^, and the sample moved at 0.06 mm s^–1^ in the *x*-axis direction in steps of 200 μm along the *y*-direction under the nano-DESI probe using a motorized *X*–*Y*–*Z* stage (Zaber Technologies Inc., Vancouver, BC) controlled by a custom-designed LabVIEW program.^32^

### Determination of Na in Rat Brain Homogenate Using ICP-AES

Two aliquots from a homogenate prepared from a rat brain were analyzed. For each sample, an amount of homogenate (19–25 mg) was dissolved in a known volume of the same solvent that was used for the nano-DESI MSI analysis of NaCl-spiked mimetic tissue model (see above). The samples were sonicated for 60 min until a clear solution was obtained, diluted 20 times using 10% HCl, and the sodium content was determined using ICP-AES (PerkinElmer DV4300 Cirros CCD) by measuring emission at 589.592 nm. Sodium was determined in blank samples that were prepared by using the solvent used for dissolving the rat brain homogenate.

### Sampling and Imaging with Pneumatically Assisted Nano-DESI

The pneumatically assisted nano-DESI probe was constructed based on the design of Duncan et al.^33^ Briefly, two fused silica capillaries (50 × 150 μm, ID × OD, Polymicro Technologies, L.L.C., Phoenix, USA) were positioned at a fixed angle (≈90°) and nitrogen gas was supplied at ≈2.5 bar. The solvent was propelled using a syringe pump (Legato 180, KD Scientific, Holliston, USA) at a flow rate of 0.5 μL min^–1^.

For method development, the nano-DESI probe was used to sample solutions from a small polypropylene cap for sampling, ionization, and acquisition of ≈50 continuous scans of a stable signal. The nano-DESI solvent was composed of methanol containing 0.1 μM dibenzo-18-crown-6 (db18c6), 0.1 μM 12-crown-4 (12c4), 0.1 μM 15-crown-5 (15c5), 0.1 μM benzo-15-crown-5 (b15c5), 25 μM d-glucose-6,6-*d*_2_ (glucose-*d*_2_), and 0.1 μM acetylcholine chloride (*N*,*N*,*N*-trimethyl-*d*_9_) (acetylcholine-*d*_9_). For evaluation of total salt concentration, the solution in the cap had the same composition as the nano-DESI solvent with the addition of various concentrations of Na^+^ and K^+^ in the form of chloride salts. For evaluation of crown ether concentrations, the solution in the nano-DESI solvent and the cap was the same with different concentrations of crown ethers (db18c6, 12c4, 15c5, and b15c5) and the cap also contained various amounts of Na^+^ and K^+^.

For surface sampling from rat brain tissue, the nano-DESI solvent contained 0.1 μM db18c6, 0.1 μM 12c4, 0.1 μM 15c5, 0.1 μM b15c5, 0.1 μM acetylcholine-*d*_9,_ and 25 μM glucose-*d*_2_ in methanol/water 9:1 v/v. The sample was moved at a speed of 0.02 mm s^–1^ under the nano-DESI probe.

Imaging of MCAO mouse brain tissue with pneumatically assisted nano-DESI was achieved using the following solvent compositions: 0.1 μM db18c6, 0.1 μM 12c4, 0.1 μM 15c5, 0.1 μM b15c5, 1.5 μM lysophosphatidylcholine 19:0 (LPC 19:0), 25 μM glucose-*d*_2_, 10.15 μM 4-aminobutyric-2,2-*d*_2_ (GABA-*d*_2_), 0.1 μM acetylcholine-*d*_9_, 1.5 μM phosphatidylcholine 12:0–13:0 (PC 25:0), 9.94 μM dl-glutamic-2,4,4-*d*_3_ acid (glutamic-*d*_3_), and 12,000-times diluted cell free ^15^N-labeled amino acid mixture in pure methanol. Individual concentrations of the ^15^N-labeled amino acids are provided in Table S1. The sample was moved at a speed of 0.04 mm s^–1^ along the *x*-axis, and each acquired line was spaced by 75 μm along the *y*-axis. Considering the scan rate of the mass spectrometer (1.7 scans s^–1^), the final pixel size was ≈24 × 75 μm^2^.

### Mass Spectrometry

All data were recorded using a QExactive mass spectrometer (Thermo Fischer Scientific, Bremen, Germany) operated at a resolution of 140,000 (*m*/Δ*m* at *m*/*z* 200), orbitrap AGC target of 1 × 10^6^, S-lens RF level of 50, heated ion transfer capillary temperature of 300 °C, and electrospray voltage of 3.2 kV.

### Data Processing

Data files were converted from RAW files to centroided mzXML using ProteoWizard MSConvert.^34^ All data processing was done in MATLAB R2019b (MathWorks, USA) using in-house scripts for obtaining the intensity of molecules of interest by locating the closest peak that is within a mass accuracy tolerance of 5 ppm. Region of interest (ROI) analysis was conducted with in-house scripts using MATLAB. ROI analysis of Na^+^-spiked mimetic tissue model was facilitated by using a mask that enabled the selection of pixels corresponding to tissue and exclude empty spaces such as holes. Details of this procedure are provided in the Supporting Information. Furthermore, values of zero intensity were excluded from the analysis, and to remove outliers, only the data between the 5th and 95th percentiles were kept. Weighted least squares regression was conducted using R in RStudio (The R Project for Statistical Computing, http://www.r-project.org). Statistical significance of mean concentrations between the healthy and ischemic areas of MCAO mouse brain tissue sections was assessed using the two-tailed Student *t* test.

## Results and Discussion

### Crown Ether Evaluation

**Figure 1:**
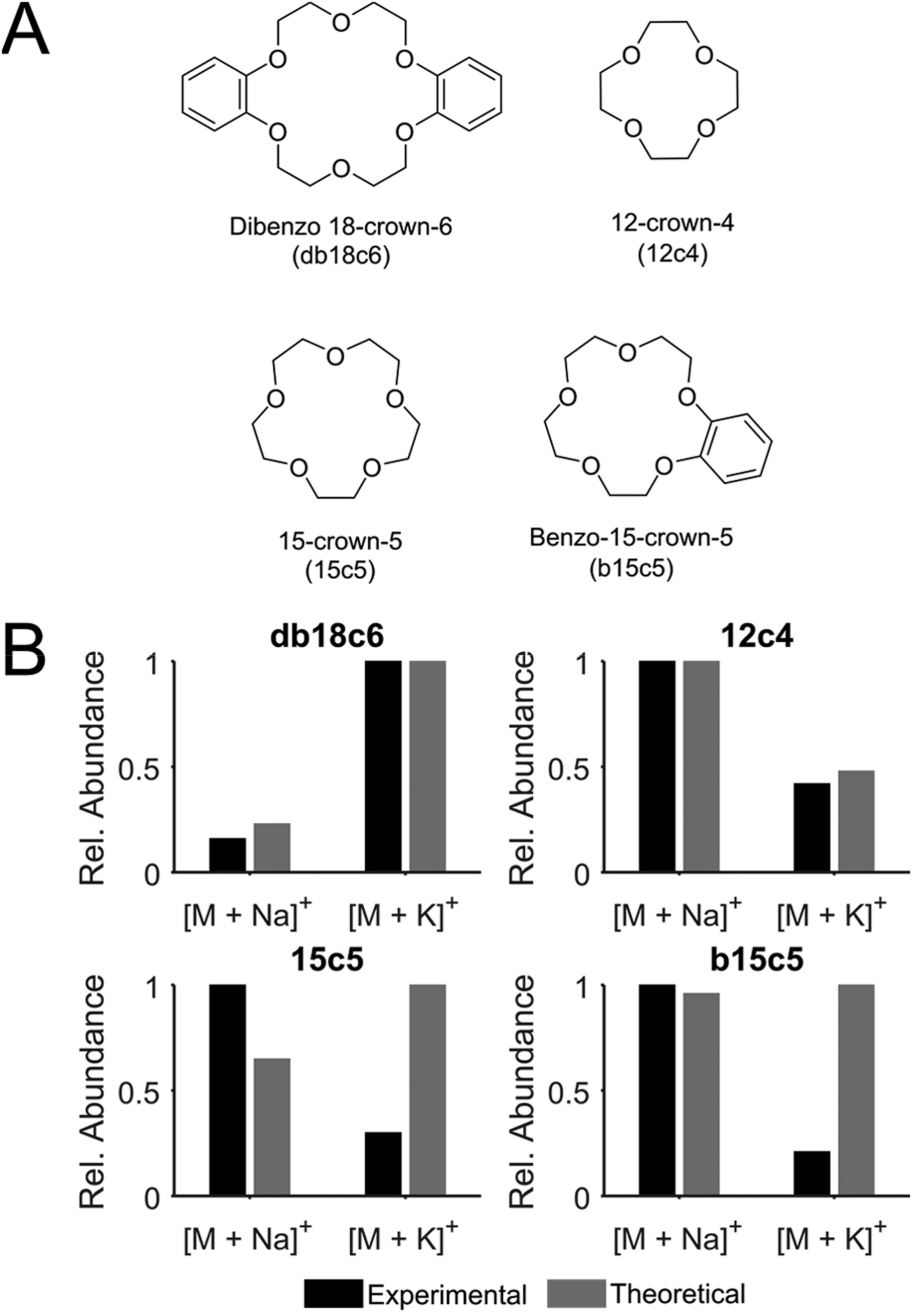
Four crown ethers evaluated as hosts. (A) Structures and nomenclature. (B) Experimental (black bars) and theoretical (gray bars) relative abundances of Na^+^ and K^+^ adducts of the respective crown ethers. The same concentrations of crown ethers, Na^+^, and K^+^ were used to obtain both experimental and theoretical data.

Crown ethers generally have a high binding affinity for Na^+^ and K^+^.^35^ However, the specific affinities depend on the individual crown ether’s structure, including their cavity size, number of electronegative oxygen atoms, and inductive side groups.^36^ For example, the larger cavity ring of db18c6 increases its affinity for the larger K^+^ over Na^+^, while the opposite is true for 12c4 with a smaller cavity size (Figure 1A, Table S2). The applicability of the four crown ethers db18c6, 12c4, 15c5, and b15c5 was evaluated in neat solutions containing 550 μM NaCl, 550 μM KCl, and 0.1 μM of each of the crown ethers using pneumatically assisted nano-DESI MSI, and their experimentally obtained relative abundances of Na^+^ and K^+^ adducts were compared to the theoretically calculated relative abundances (see the Supporting Information for details on calculations) (Figure 1B). The results show excellent agreement between the experimental and theoretical data for db18c6 and 12c4, which indicates that the theoretical binding affinities are sustained through electrospray ionization. In contrast, 15c5 and b15c5 show quite large discrepancies, which are due to isobaric overlaps between system peaks and the sodiated crown ether adducts at *m*/*z* 243.12 and 259.12. These overlaps cause an increased intensity of the Na^+^ adducts and thereby shift the relative abundances of their respective Na^+^ and K^+^ adduct, thereby excluding them as hosts. Although this is not the case for the 12c4 crown ether, it was instead excluded because its smaller ring cavity size enables the formation of protonated adducts (Figures S1 and S2). The formation of protonated adducts, in addition to Na^+^ and K^+^ adducts, will split the signal and make the Na^+^ and K^+^ adduct formation sensitive to pH changes. Consequently, db18c6 was selected as the preferred crown ether host because no protonated adducts were detected (Figures S1 and S2) and there was a good agreement between the experimental and theoretical relative abundances of Na^+^ and K^+^ adducts (Figure 1B), indicating minimal spectral overlaps for these mass channels. Further, the addition of db18c6 to the nano-DESI solvent neither interferes with the detection of endogenous metabolites and lipids (Figure S3) nor disturbs the alkali metal adduct distribution of lipids (Figure S4). Thus, db18c6 is suitable for use in nano-DESI MSI of tissue sections.

### Quantification at Varying Salt Concentrations

Although db18c6 is suitable, the host–guest method also needs to provide quantitative results to determine the impact of Na^+^ and K^+^ in biological systems. In particular, the acquired relative intensities need to mirror the actual relative abundance of each cation, and it needs to be independent of the total salt concentrations in the sample. Therefore, first, neat solutions containing different proportions of Na^+^ and K^+^, in equally spaced increments of 20% ranging from 10% Na^+^–90% K^+^ to 90% Na–10% K^+^, were analyzed with direct infusion ESI at a total salt concentration of 200 μM (Figure S5). These results show, both experimentally and theoretically, that the higher binding affinities of db18c6 for K^+^ (log *K*_b_ is 5.0 for K^+^ and 4.37 for Na^+^) cause nonlinear correlations between the detected adduct intensities and the alkali metal ion concentrations. These nonlinear correlations make it difficult to directly determine the individual Na^+^ and K^+^ concentrations based on their complexation with db18c6 (Figure S5A,B,D,E). Specifically, the higher affinity of db18c6 for K^+^ in combination with the higher amount of K^+^ in the brain causes the [db18c6 + K]^+^ signal to become saturated (Figure S6). Therefore, we use the adduct intensity ratio, determined as [M + Na]^+^ divided by [M + K]^+^, to remove effects of equilibrium concentration saturation and allow for comparison of Na^+^ and K^+^ using linear regression (Figure S5C,F).

**Figure 2:**
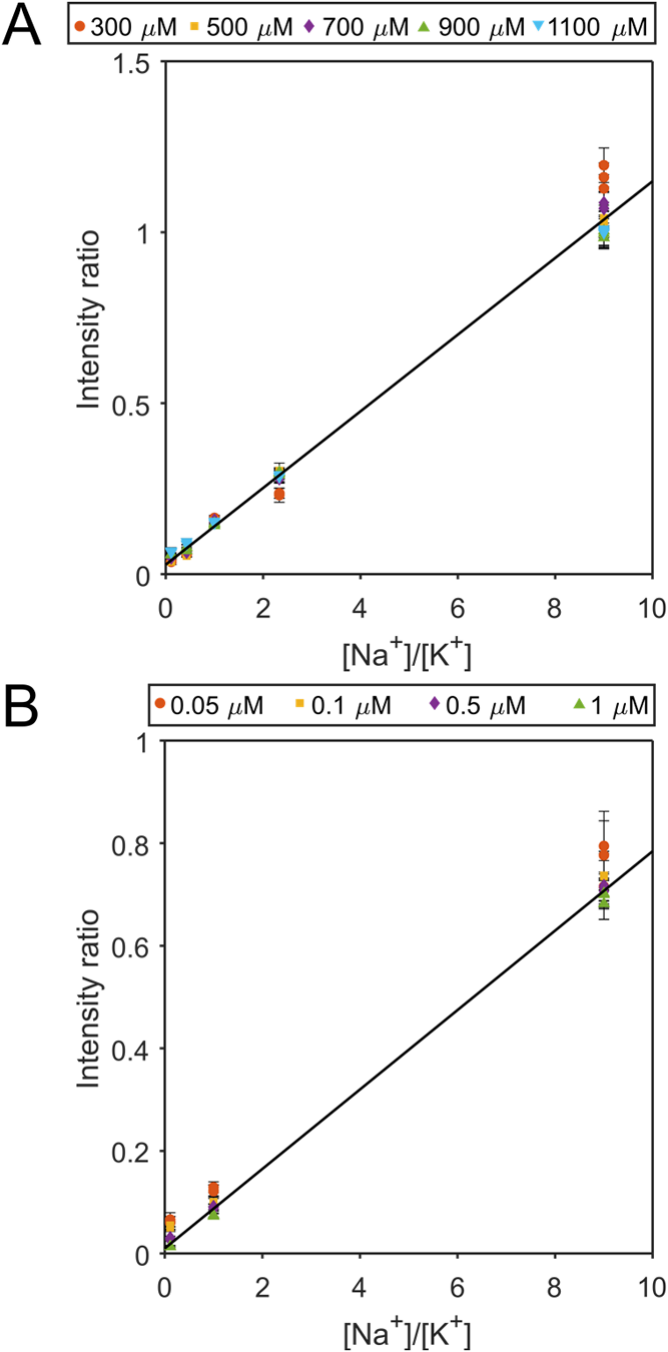
Intensity ratio is independent of total salt and crown ether concentrations. (A) Weighted least squares of Na^+^/K^+^ adduct intensity ratio of db18c6 vs [Na^+^]/[K^+^] with 1/σ^2^ used as weighting at various total salt concentrations. The fitted line has the linear equation *y* = 0.112*x* + 0.028 with *R*^2^ = 0.9738. At each concentration ratio, three replicates for each total salt concentration condition were acquired. Error bars show the standard deviation of each sample (scan-to-scan variation). (B) Na^+^/K^+^ adduct intensity ratio of db18c6 vs [Na^+^]/[K^+^] at different crown ether concentrations. The fitted line has the linear equation *y* = 0.077*x* + 0.001 with *R*^2^ = 0.9603. At each individual concentration, three replicates were acquired. Error bars represent one standard deviation of each replicate (scan-to-scan variation).

Second, the complexation of db18c6 with Na^+^ and K^+^ was evaluated using five different total salt concentrations of 300, 500, 700, 900, and 1100 μM of [NaCl] + [KCl] and different proportions of Na^+^ and K^+^ (10% Na^+^–90% K^+^ to 90% Na–10% K^+^) at each total salt concentration, using pneumatically assisted nano-DESI (Table S3). The results in Figure 2A depict negligible differences for the various total salt concentrations studied on the Na^+^/K^+^ adduct intensity ratio, showing that the ratio is not affected by differences in the amount of salt. The increased residuals at higher proportions of Na^+^ result from higher individual intensities and heteroscedastic noise, as observed from larger standardized residuals at larger values of the *x*-variable (Figure S7);^37^ thus, the data were fitted using weighted least squares. Third, we show that the obtained linear regression can be used to determine unknown [Na^+^]/[K^+^] at small biases of only 4–13% (Figure S8 and Table S4). Collectively, the results show that the crown ether db18c6 can be successfully used as a host to quantitatively determine various [Na^+^]/[K^+^] in total salt concentrations ranging between 200 and 1100 μM.

### Independence of Crown Ether Concentrations

The independence of amenable crown ether concentrations on quantification using the host–guest method is necessary for robustness and flexibility, both for sample types and mass spectrometry operating conditions. Therefore, neat solutions at a total salt concentration of 500 μM and with varying proportions of Na^+^ and K^+^ (10% Na^+^–90% K^+^, 50% Na^+^–50% K^+^, and 90% Na^+^–10% K^+^) were analyzed with four different db18c6 concentrations: 0.05, 0.1, 0.5, and 1 μM. The results show that the Na^+^/K^+^ adduct intensity ratios are conserved for all crown ether concentrations (Figure 2B), demonstrating that any db18c6 concentration between 0.05 and 1 μM could be used to monitor [Na^+^]/[K^+^] changes. This displays the flexibility and robustness of the host–guest method to be used in different applications and with a range of instrumental conditions that may require higher or lower crown ether concentrations.

### Compatibility with High Molecular Complexity

**Figure 3:**
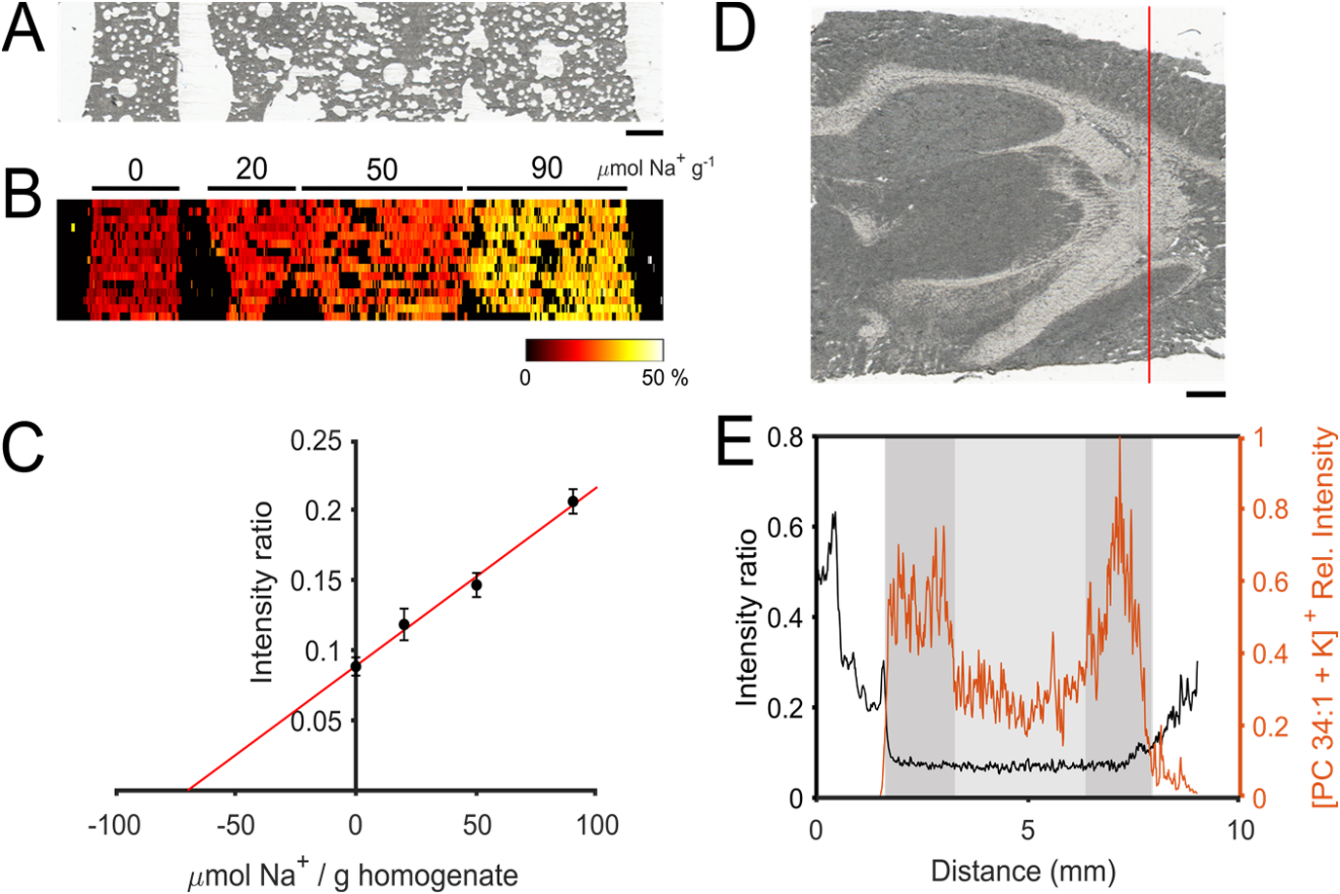
Sampling and imaging of Na^+^ and K^+^ from tissue sections. (A) Optical image of the Na^+^-spiked mimetic tissue model section, starting with a blank and then increasing Na^+^ concentrations from left to right. (B) Image showing the db18c6 Na^+^/K^+^ adduct intensity ratio (from 0 to 50% relative intensity ratio) obtained after nano-DESI MSI of the section shown in (A). (C) Extracted ROI data from each layer of the analyzed section of the mimetic tissue model showing the average Na^+^/K^+^ adduct intensity ratio of db18c6 vs the spiked Na^+^ amount in each layer. Error bars show one standard deviation of each ROI. The red line indicates a linear fit through the data points with equation *y* = 0.00127*x* + 0.089. (D) Optical image of the analyzed rat brain tissue section where the red line indicates the acquired line scan. (E) Na^+^/K^+^ adduct intensity ratio of db18c6 (left axis, black line) and relative intensity of endogenous [PC 34:1 + K]^+^ (right axis, orange line) measured during the sampling of the line shown in (A) after using a moving average algorithm (*n* = 3). Gray shaded areas indicate sampling from gray matter regions, while the lighter gray area indicates the white matter region of the brain tissue section. Areas with no shading indicate sampling from the glass surface. Scale bars in (A,D) show 1 mm, and the color scale in (B) refers to relative intensity ratio.

The molecular complexity of samples such as tissue could cause interferences and produce artifacts during ionization, ultimately affecting the accuracy of quantification.^12^ To assess this, a mimetic brain tissue model was constructed and spiked with increasing amounts of Na^+^ in different segments. Quantification of the non-spiked segment was then performed by standard addition using pneumatically assisted nano-DESI MSI with 0.1 μM db18c6 doped in the solvent (Figure 3A,B). Then, the Na^+^/K^+^ adduct intensity ratio data were extracted from each segment of the mimetic tissue and plotted against the Na^+^ concentrations of the respective segments. The results show a linear correlation between signal and Na^+^ concentration despite the high chemical complexity (Figures 3C and S9), indicating the feasibility for quantification with our method. Furthermore, by considering the obtained data as a standard addition curve, we could determine the Na^+^ concentration in the non-spiked layer to be 70 ± 6 μmol g^–1^ (Figure 3C). The accuracy of this result was further assessed by parallel analysis using ICP-AES. Specifically, we established an external calibration curve and analyzed the Na^+^ in two aliquots of a rat brain homogenate with ICP-AES. The ICP-AES analysis determined the Na^+^ concentration to be 55 ± 14 μmol g^–1^, which is in good agreement with the results from our new host–guest method. Similar results obtained via the two extremely different analytical approaches demonstrate the feasibility of our host–guest approach to indirectly determine the concentration of Na^+^ in chemically complex tissue sections using MSI.

In addition to monitoring Na^+^/K^+^ changes in homogeneous tissue sections, the method needs to be accurate in dynamic molecular compositions. Two morphologically different brain tissue regions are white matter, which has a high abundance of glycolipids, and gray matter, with a high abundance of phospholipids.^12^ Therefore, a tissue transect was performed using pneumatically assisted nano-DESI MS with 0.1 μM db18c6 doped in the solvent over both gray and white matter areas in a brain tissue section (Figure 3D). In Figure 3E, the Na^+^/K^+^ adduct intensity ratio for db18c6 is shown (black line) along with the intensity of the endogenous phospholipid [PC 34:1 + K^+^] (orange line). Three different regions are highlighted in Figure 3E; white areas are glass, gray areas are gray matter, and the light gray area corresponds to the white matter. Further, it was observed that as the nano-DESI probe comes into contact with the tissue surface, the individual mass spectrometric signals of [db18c6 + Na]^+^ and [db18c6 + K]^+^ are suppressed due to the high abundance of phospholipids present in the gray matter (Figure S10). As can be seen in Figure 3E, the intensity of the endogenous phospholipid PC 34:1 decreases when the white area is sampled as expected. Importantly, despite the alterations in molecular composition between the white and the gray matter, the Na^+^/K^+^ adduct intensity ratio remains consistent (Figure 3E). This was expected considering that the matrix, that is, tissue environment, would affect the sodiated and potassiated adducts to the same extent, and therefore, monitoring the Na^+^/K^+^ adduct intensity ratio when Na^+^ and K^+^ are in vast excess conveniently removes such matrix effects. However, a simultaneous decrease in both ions would be difficult to distinguish using the ratio. Collectively, these results demonstrate that the method is also applicable in tissue samples with a highly dynamic chemical environment.

### Quantitative Imaging

**Figure 4:**
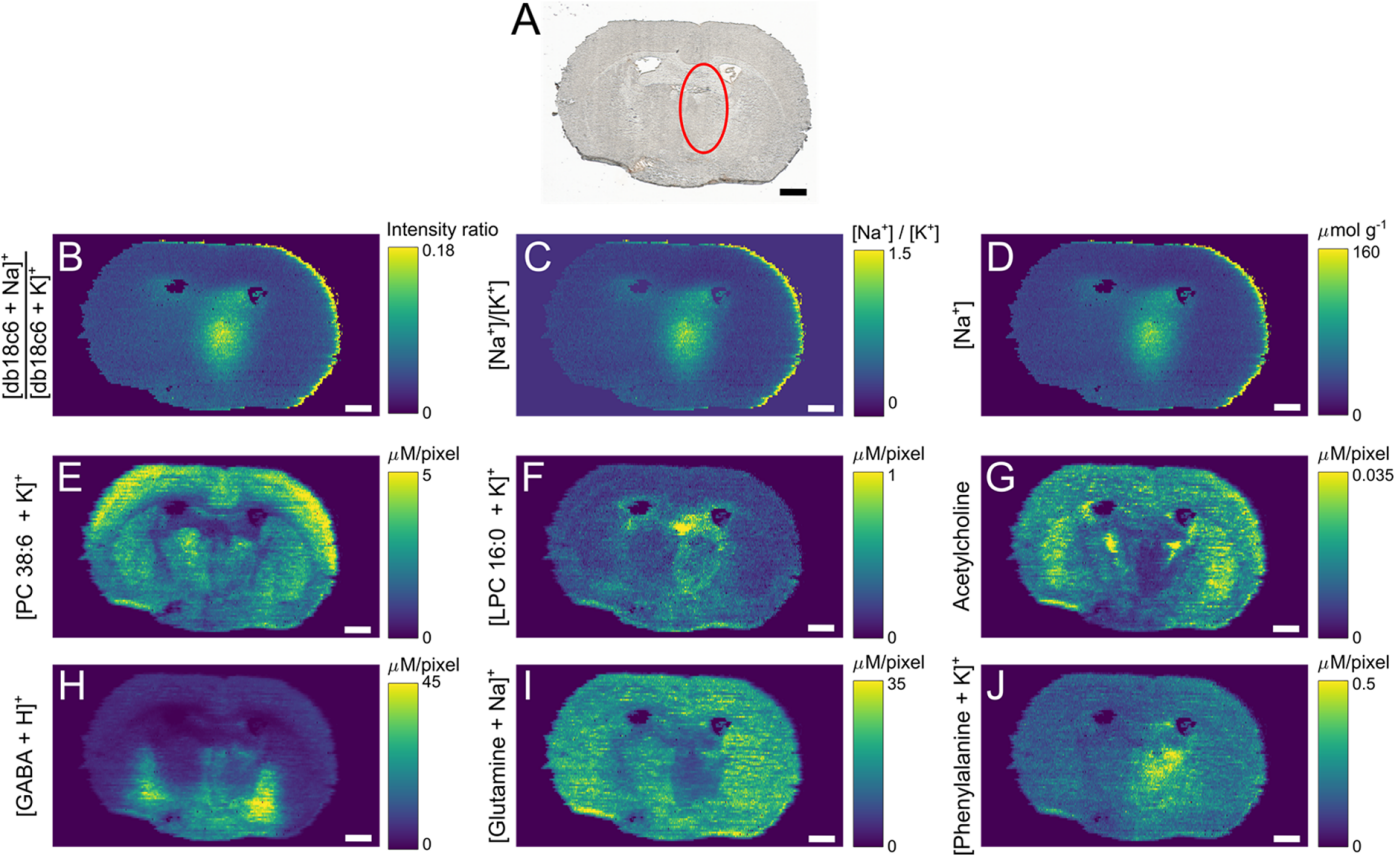
Combined molecular and elemental information obtained with nano-DESI MSI. (A) Optical image of the MCAO mouse brain section, where the ischemic region is marked with a red circle, images of (B) Na^+^/K^+^ adduct intensity ratio of db18c6, (C) concentration ratio Na^+^/K^+^ obtained using the regression line of Figure 2A and data from (B), (D) Na^+^ concentration obtained using the regression line of Figure S13 and data from (B), (E) [PC 38:6 + K]^+^ normalized to [PC 25:0 + K]^+^, (F) [LPC 16:0 + K]^+^ normalized to [LPC 19:0 + K]^+^, (G) acetylcholine normalized to acetylcholine-*d*_9_, (H) [GABA + H]^+^ normalized to [GABA-*d*_2_ + H]^+^, (I) [glutamine + Na]^+^ normalized to [glutamine-^15^N_2_ + Na]^+^, and (J) [phenylalanine + K]^+^ normalized to [phenylalanine-^15^N + K]^+^. Scale bars show 1 mm. The maximum color scale has been adjusted to increase the clarity of less intense features.

One such environment is ischemic stroke, and it is well known that ischemic stroke significantly alters the tissue distribution of Na^+^, K^+^, and small molecules.^1,38−41^ Quantitative distributions of Na^+^ and K^+^ can be obtained with microdissection followed by ICP-AES; however, this does not provide simultaneous metabolite information. Here, an MCAO mouse brain tissue section, with the ischemic region circled in red, was imaged with both db18c6 and internal standards included in the nano-DESI for simultaneous quantitative elemental and molecular imaging (Figure 4A). The Na^+^/K^+^ adduct intensity ratio of db18c6 is clearly elevated levels in the ischemic area (Figures 4B and S11). Note that the ion images in Figure 4B–J depict only those pixels that are correlated with endogenous PC 34:1 because the Na/K adduct intensity ratio of db18c6 is also visible outside the tissue (Figure S11). Interestingly, the distribution of Na^+^/K^+^ is heterogeneous in the ischemic area with a higher ratio in the center compared to the edges, which mirrors our previous observation of the distribution of LPC 18:1 in the damaged area and suggests a deeper damage in the center.^39^ By applying the obtained regression line (Figure 2B) to each pixel, the Na^+^/K^+^ adduct intensity ratio of db18c6 was converted to [Na^+^]/[K^+^]. The average [Na^+^]/[K^+^] was 0.84 ± 0.06 in the ischemic ROI and 0.29 ± 0.08 in the healthy ROI, showing that the concentration ratio is about 3 times higher in the damaged area than in the corresponding healthy hemisphere (Figures 4C and S12). Further, because both the Na^+^ concentration and the extraction efficiency of the mimetic tissue model (Figure 3A–C) and the mouse brain tissue section (Figure 4A) are expected to be similar, the previously quantified concentration of 70 ± 6 μmol g^–1^ of Na^+^ can be used as a measure for the healthy tissue. Thus, the Na^+^/K^+^ adduct intensity ratio can be further converted to [Na^+^] to show that the ischemic region has around 150 μmol g^–1^ of Na^+^ in the center (Figures 4D and S13). Using the average Na^+^/K^+^ adduct intensity ratio of db18c6 for the ischemic and the healthy region obtained after analysis of three tissue sections, we determine that the average Na^+^ concentration in the larger ischemic area is 96 μmol g^–1^, while in the healthy area, it is 48 μmol g^–1^ (Table 1). Subsequently, we can also determine the [K^+^] because the [Na^+^]/[K^+^] and the [Na^+^] are known. In the ischemic region, we determine the K^+^ concentration to be 115 μmol g^–1^, and in the healthy region, we determine it to be 169 μmol g^–1^ (Table 1), showing that [Na^+^] is increasing and [K^+^] is decreasing in the ischemic region of the MCAO mouse brain. Notably, according to previous studies, [K^+^] is around 10 μmol g^–1^ higher in the gray matter than in the white matter, but this is below our sensitivity (depicted as the standard deviation of our measurement in Table 1) and therefore not observed.^42,43^ The results of our concentrations were further verified using ICP-AES analysis, in which the concentrations in healthy rat brain tissue were determined to be 55 ± 14 μmol g^–1^ of Na^+^ and 99 ± 29 μmol g^–1^ of K^+^. Our findings are consistent with the expected stroke pathophysiology^1,2^ as well as previous reports.^44,45^ In particular, Mulder et al. observed increased Na^+^/K^+^ in the ischemic region using SIMS in a transient MCAO mouse model,^44^ and Young et al. reported quantitative alterations of sodium and potassium ions in different rat brain regions at various time points after MCAO.^45^ Specifically, Young et al. determined 53 μmol g^–1^ of Na^+^ and 112 μmol g^–1^ of K^+^ in brain tissue of unoperated rats.^45^ Although there is a small discrepancy between the potassium concentrations for the healthy region determined with our method compared to Young et al. and our ICP-AES, this may be the result of comparing rat and mouse brain tissue. Furthermore, rat brain homogenates were also used to construct the calibration sample and report results of mouse brain tissue in our method; thus, potential differences among the animals might have contributed to accurate concentration discrepancies. Overall, the results show that quantitative elemental MSI is possible in the complex stroke model using our developed host–guest chemistry.

**Table 1:** Average^a^ Detected Concentrations (±1 Standard Deviation^a^) Extracted from ROI Analysis of Three Independent MCAO Mouse Brain Tissue Sections Showing Differences among the Ischemic and the Healthy Areas

		detected concentration	
		ischemic area	healthy area
μmol g^–1^	[Na^+^]^b^	96 ± 5	48 ± 7
	[K^+^]^b^	115 ± 2	169 ± 17
μM/pixel	PC 38:6^b^	1.56 ± 0.10	1.86 ± 0.12
	LPC 16:0^b^	0.47 ± 0.03	0.33 ± 0.03
	acetylcholine^b^	0.0097 ± 0.0013	0.0134 ± 0.0009
	GABA^b^	11.41 ± 0.52	7.95 ± 0.48
	glutamine	12.85 ± 1.74	17.32 ± 1.70
	phenylalanine^b^	0.28 ± 0.01	0.15 ± 0.01

a Means of each ROI from all technical replicates (*n* = 3) were combined, and standard deviations were calculated from the pooled ROI means.

b Statistically significant means (*p* < 0.05) among the healthy and ischemic areas using the two-tailed Student *t* test.

In addition to Na and K, selected molecular ion images are displayed in Figure 4E–J. Note that all color scales are quantitative and individual for each ion image by use of internal standards^46^ and summarized in Table 1. Figure 4E depicts the distribution of PC 38:6, which exhibits minimal impact by the stroke event and therefore mainly shows different morphological regions of the mouse brain section. On the contrary, ischemia-mediated molecular alterations, such as the increased production of LPC 16:0, is visible in the damaged area (Figure 4F), which is consistent with previous reports.^39,40,47,48^ In addition, alterations in neurotransmitters and amino acids distributions are observed. Specifically, in the ischemic region, acetylcholine showed decreased levels (Figure 4G), GABA was increased (Figure 4H), glutamine was decreased (Figure 4I), and phenylalanine was increased (Figure 4J). These findings support our method’s capability of quantification of metabolites clearly perturbed by ischemia and simultaneous quantification of spatially resolved individual concentrations of Na^+^ and K^+^. We envision that the depth of information that can be acquired through the combination of our developed host–guest method and quantitative spatial metabolomics will provide new insights into metabolic pathways even in severely chemically altered tissue environment like ischemia.

## Conclusions

Here, we present a simple method for visualization and quantitative determination of changes in [Na^+^] and [K^+^] using host–guest chemistry with crown ethers doped in the solvent for nano-DESI MSI of thin tissue sections. We evaluated the use of different crown ethers for revealing quantitative changes in [Na^+^]/[K^+^] and found db18c6 to be the most robust. The crown ether db18c6 was doped in the nano-DESI solvent along with appropriate internal standards for simultaneous quantitative elemental and molecular MSI. The main advantage of our method is the combined and simultaneous quantification of Na^+^, K^+^, lipids, neurotransmitters, and amino acids, which enhances the information obtained from each tissue section. The method was demonstrated using a MCAO mouse stroke model and revealed that [Na^+^]/[K^+^] ratios are increased up to 2.9 times in the ischemic region. Further, individual concentrations of Na and K were determined, and a 2 times increase in [Na^+^] and 1.5 times decrease in [K^+^] were determined in the ischemic region. In addition, accumulation and depletion of several classes of metabolites in the ischemic region were detected, when compared to the healthy brain region. We envision that our method will be easily adapted for future MSI applications, where disease progression and identification-associated metabolic pathways are monitored for basic understanding and for future treatment targets.

## Supporting Information Available

Preparation of NaCl-spiked mimetic tissue model, programmatic exclusion of tissue holes, extraction of ROI data, calculation of theoretical equilibrium concentrations for crown ether adducts with Na^+^ and K^+^, concentrations of ^15^N-labeled amino acids in the nano-DESI solvent, protonated adduct signals for crown ethers in neat solution and on tissue, mass spectrum of mouse brain tissue during nano-DESI with db18c6-doped solvent, lipid adduct distribution during sampling with nano-DESI, binding affinities of crown ethers with Na and K ions, theoretical and experimental individual abundances of [db18c6 + Na]^+^ and [db18c6 + K]^+^, ion images of individual [db18c6 + Na]^+^ and [db18c6 + K]^+^, concentrations of Na^+^ and K^+^ used for method development, db18c6 data heteroscedasticity, db18c6 direct infusion ESI-MS regression line, expected and predicted [Na^+^]/[K^+^] in tested solutions, ion images for ROI selection in mimetic tissue model, db18c6 mass spectrometric traces on tissue, unprocessed image of Na^+^/K^+^ adduct intensity ratio for db18c6, [Na^+^]/[K^+^] obtained from three MCAO mouse brain tissue sections, and calibration line using the mimetic tissue model (PDF)
