# Supplementary material for: Host–Guest Chemistry for Simultaneous Imaging of Endogenous Alkali Metals and Metabolites with Mass Spectrometry
Source: Anal Chem. 2022 Jan 25;94(5):2391–8. doi: 10.1021/acs.analchem.1c03913 (PMC8829828; doi:10.1021/acs.analchem.1c03913)
Supplement: Supplementary file 1 — ac1c03913_si_001.pdf [file ac1c03913_si_001.pdf]

Supporting Information

Host-guest chemistry for simultaneous imaging of endogenous alkali metals and metabolites with mass spectrometry

Leonidas Mavroudakis, Kyle D. Duncan†, Ingela Lanekoff*

*Corresponding Author

Ingela Lanekoff - Department of Chemistry – BMC, Uppsala University, Uppsala 75124, Sweden; orcid.org/0000-0001-9040-3230 Email: ingela.lanekoff@kemi.uu.se

Authors

Leonidas Mavroudakis - Department of Chemistry – BMC, Uppsala University, Uppsala 75124, Sweden; orcid.org/0000-0002-6520-3437

Kyle D. Duncan - Department of Chemistry – BMC, Uppsala University, Uppsala 75124, Sweden; orcid.org/0000-0003-0575-0858

Present Addresses

†Kyle D. Duncan – Department of Chemistry, Vancouver Island University, British Columbia, V94 5S5, Canada

Table of Contents

Preparation of NaCl spiked mimetic tissue model..3

Exclusion of tissue holes after imaging and ROI of NaCl spiked mimetic tissue model...3

Extraction of ROI data from NaCl spiked mimetic tissue model 3

Calculation of theoretical equilibrium concentrations for crown ether adducts with Na^+ and K^+..4

Table S1..5

Figure S1 6

Figure S2 7

Figure S3 8

Figure S4 8

Table S2..9

Figure S5 9

Figure S6 9

Table S3..10

Table S4..10

Figure S7 11

Figure S8 11

Figure S9 12

Figure S10 13

Figure S11 13

Figure S12 14

Figure S13 14

Preparation of NaCl spiked mimetic tissue model

Two rat brains were chopped, and pieces were distributed into 2 mL tubes containing 1.4 mm ceramic spheres (Lysing Matrix D, MPBio) for homogenization with a bead homogenizer (FastPrep-24, MPBio) at 4 m s^{-1} (30 s) and for 2-3 rounds. All homogenates were combined into one tube, the pooled homogenates were aliquoted and the mass of each aliquot was recorded (0.227 – 0.284 g). For spiking each aliquot of homogenate, a concentrated aqueous NaCl solution was added to a final Na^+ concentration of 20, 50 and 90 µmol g^{-1}. The amount of spiked solution in each tissue homogenate was below 2 % v/v. One aliquot of brain homogenate was not spiked with NaCl. To differentiate the different layers during mass spectrometric analysis, non-endogenous markers were spiked at known concentrations. Specifically, the non-spiked layer contained 24.5 μM of diclofenac, the 20 μmol g^{-1} Na^+ layer had 28.2 μM of enalapril, the 50 μmol g^{-1} Na^+ layer had 55.7 μM phosphatidylethanolamine (PE) 30:0, and the 90 μmol g^{-1} Na^+ layer had 33.8 μM of GABA-^{15}N. The amount of spiked solution in each tissue homogenate was below 0.8 % v/v. Although mass spectrometric signals from diclofenac and PE 30:0 were not observed, the detection of enalapril and GABA-^{15}N enabled the region of interest analysis of all layers in the mimetic tissue model. All homogenates; spiked and non-spiked; were individually mixed in the bead homogenizer for 30 s at 4 m s^{-1}. The homogenates were stored at -80 °C until use. The mimetic tissue model, containing different layers of NaCl-spiked brain homogenate, was prepared by using a 3 mL plastic syringe which was modified and used as a mold. First, the non-spiked homogenate was added carefully in the syringe mold (≈ 300 μL) and immediately frozen in dry-ice chilled ethanol. Then, the syringe mold was taken out of the cold ethanol and quickly the lowest NaCl spiked homogenate was added on top of the previous homogenate layer, dipped in cold ethanol for freezing and the process is repeated until all layers have been added. Care was taken for the layers not to mix with each other during the process which can be caused if they start to warm up. This resulted in a mimetic tissue model where the lowest layer is the non-spiked followed by the 20, 50 and 90 μmol NaCl g^{-1}.

Exclusion of tissue holes after imaging and ROI of NaCl spiked mimetic tissue model

Tissue irregularities caused during the preparation of the mimetic tissue model i.e. holes, were removed programmatically using a mask that differentiates tissue and non-tissue areas. This mask was created by selecting pixels from the optical image of the tissue section that have summed red-green-blue (RGB) triplet less than 700. This threshold value was determined empirically and was based on the observation that the tissue area and the glass area (i.e. non-tissue area) have distinct differences in their RGB triplets. Subsequently, for image generation and ROI analysis only data that corresponded to tissue area were considered.

Extraction of ROI data from NaCl spiked mimetic tissue model

To further assess the accuracy of the obtained intensity ratio for quantitative analysis of [Na^+], region of interest (ROI) analysis was performed. The ion image of endogenous [LPC 16:0 + Na]$^+$ normalized to [LPC 19:0 + Na]$^+$ is shown in Figure S9A to demonstrate observed tissue holes. These holes will impact the variation of the extracted ROI data and thus they were excluded during the analysis, as described in the section above. The Na^+/K^+ adduct intensity ratio image of db18c6 before removing tissue holes programmatically is shown in Figure S9B. The constant supply of db18c6 through the nano-DESI solvent makes the visualization of the different layers difficult. To facilitate ROI selection, a composite ion image of [enalapril + K]$^+$ and [GABA-^{15}N + Na]$^+$ was used as indicator of the 20 and 90 μmol g^{-1} layers, respectively

(Figures S9C, S9E & S9G). The ROI for the non-spiked layer was selected based on the distribution of endogenous [creatine + K]$^+$ (Figure S9D). Finally, the ROI for the 50 μmol g^{-1} layer was selected based on the absence of any signal in that tissue area (Figure S9F).

Calculation of theoretical equilibrium concentrations for crown ether adducts with Na$^+$ and K$^+$

Let M be the crown ether molecule, (M+Na)$^+$, (M+K)$^+$ the crown ether adducts with Na$^+$ and K$^+$, respectively, and [(M+Na)$^+$], [(M+K)$^+$] the equilibrium concentrations of the complexes with sodium and potassium ions, respectively For the calculation of [(M+Na)$^+$] and [(M+K)$^+$] we consider a system of one crown ether where both Na$^+$ and K$^+$ are present simultaneously. In the experiment presented in Figure 1B, all four crown ethers were present simultaneously in the solution along with Na$^+$ and K$^+$ ions. However, the competition for sodium and potassium ions among the crown ethers is negligible when [M] << [Na$^+$], [K$^+$].

$$M + Na^+ \leftrightarrow (M + Na)^+ \ (\mathbf{1})$$

$$M + K^+ \leftrightarrow (M + K)^+ \ (\mathbf{2})$$

For the reactions (1) and (2), in equilibrium, we can write the respective equilibrium expressions, termed as binding affinity, K_b:

$$K_{b,Na} = \frac{[(M + Na)^+]}{[M]_f[Na^+]_f} \ (\mathbf{3})$$

$$K_{b,K} = \frac{[(M + K)^+]}{[M]_f[K^+]_f} \ (\mathbf{4})$$

Where $[M]_f$ is the free crown ether concentration and $[Na^+]_f$, $[K^+]_f$ are the free sodium and potassium concentrations, respectively.

According to mass balance equations:

$$[M]_T = [M]_f + [(M + Na)^+] + [(M + K)^+] \ (\mathbf{5})$$

$$[Na^+]_T = [Na^+]_f + [(M + Na)^+] \ (\mathbf{6})$$

$$[K^+]_T = [K^+]_f + [(M + K)^+] \ (\mathbf{7})$$

Where $[M]_T$, $[Na^+]_T$ and $[K^+]_T$ are the total concentrations of crown ether, sodium and potassium, respectively.

Substitution of $[M]_f$, $[Na^+]_f$ and $[K^+]_f$ from equations (5), (6) and (7) in equations (3) and (4) results in two independent equations where [(M+Na)$^+$] and [(M+K)$^+$] can be determined provided that $[M]_T$, $[Na^+]_T$, $[K^+]_T$, $K_{b,Na}$ and $K_{b,K}$ are known. The two equations used for calculating [(M+Na)$^+$] and [(M+K)$^+$] are shown below:

$$[(M + Na)^+]^2 - \left(\frac{1}{K_{b,Na}} + [M]_T + [Na^+]_T - [(M + K)^+]\right)[(M + Na)^+] + [M]_T[Na^+]_T - [Na^+]_T[(M + K)^+] = 0 \ (\mathbf{8})$$

$$[(M+K)^+]^2 - \left(\frac{1}{K_{b,K}} + [M]_T + [K^+]_T + [(M+Na)^+]\right)[(M+K)^+] + [M]_T[K^+]_T - [K^+]_T[(M+Na)^+] = 0 \ (\mathbf{9})$$

For example, the theoretical abundances calculated in Figure 1B were obtained by using $[M]_T$ = 0.1 μM, $[Na^+]_T$ = 550 μM, $[K^+]_T$ = 550 μM and the corresponding binding affinities $K_{b,Na}$, $K_{b,K}$ for each crown ether (see Table S2). The two nonlinear simultaneous equations (8 and 9) were solved numerically using MATLAB's *vpasolve* function, which provides the solutions for unknowns ($[(M+Na)^+]$ and $[(M+K)^+]$). Equations (8) and (9) give three solutions each, however, certain solutions were discarded if the following conditions were met:

$[(M+Na)^+] > [M]_T$ *or* $[(M+Na)^+] > [Na^+]_T$ *or* $[(M+Na)^+] < 0$

$[(M+K)^+] > [M]_T$ *or* $[(M+K)^+] > [K^+]_T$ *or* $[(M+K)^+] < 0$

Table S1. Concentrations and % ^{15}N enrichment of each ^{15}N-labelled amino acid originating from the obtained 12000-times diluted cell free ^{15}N-labelled amino acids standard mixture that was added to the nano-DESI solvent as internal standards for quantitative imaging.

Amino acid	% ^{15}N enrichment	Concentration (μM)
Glutamic acid	99.3	4.00
Aspartic acid	99.3	7.00
Arginine	99.3	1.25
Histidine	99.3	0.42
Lysine	99.3	1.42
Asparagine	99.3	7.00
Cysteine	> 98.2	1.67
Glutamine	98.7	1.67
Methionine	99.3	0.67
Serine	99.3	2.42
Threonine	99.3	2.75
Phenylalanine	99.3	1.67
Tryptophan	95.5	1.67
Tyrosine	99.3	0.83
Alanine	99.3	6.25
Leucine	99.3	3.75
Isoleucine	99.3	2.33
Valine	99.3	3.00
Glycine	99.3	4.83
Proline	99.3	1.67

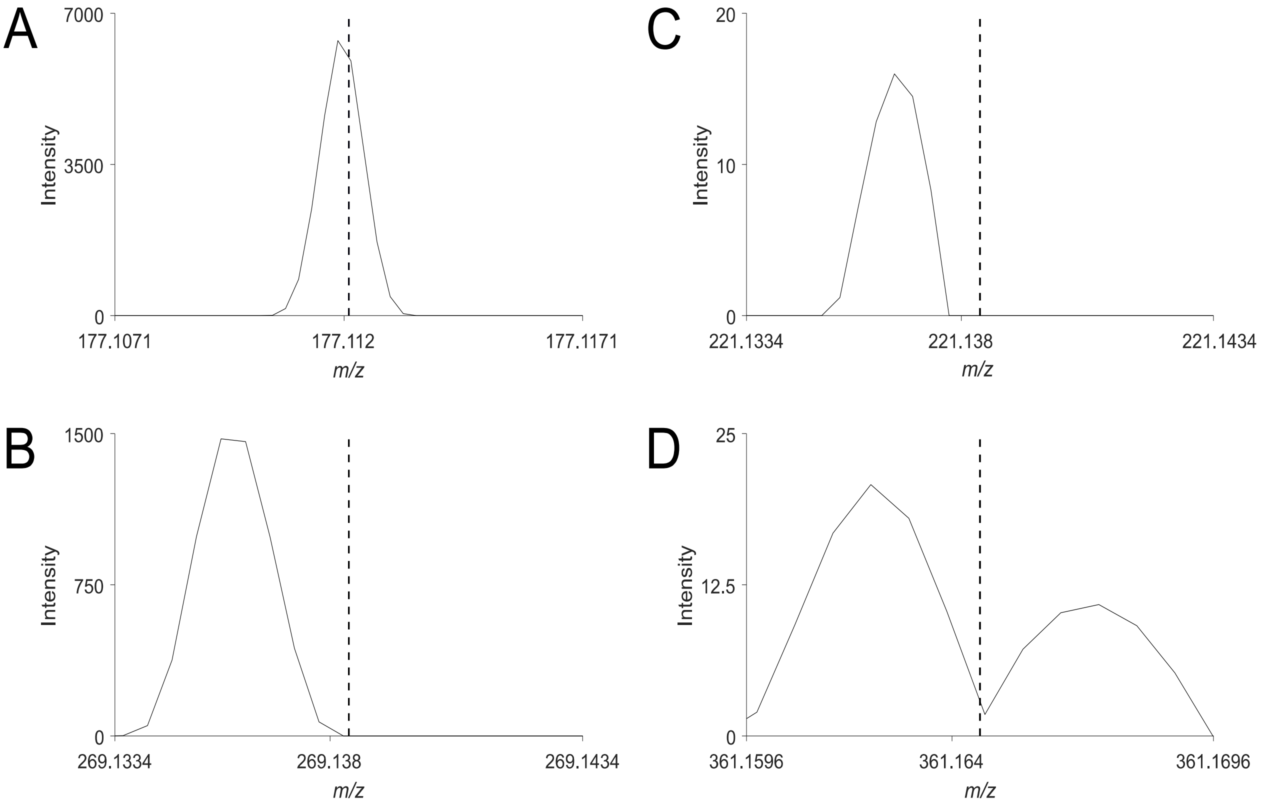

Figure S1. Expected m/z ranges for protonated adducts of the tested crown ethers; A)12c4, B) b15c5, C) 15c5, D) db18c6 when a solution containing 250 μM Na^+, 250 μM K^+ and 0.1 μM of each crown ether was analyzed with nano-DESI. Dashed lines indicate the theoretical m/z for each protonated adduct, showing that only the 12c4 in A) generates a protonated adduct.

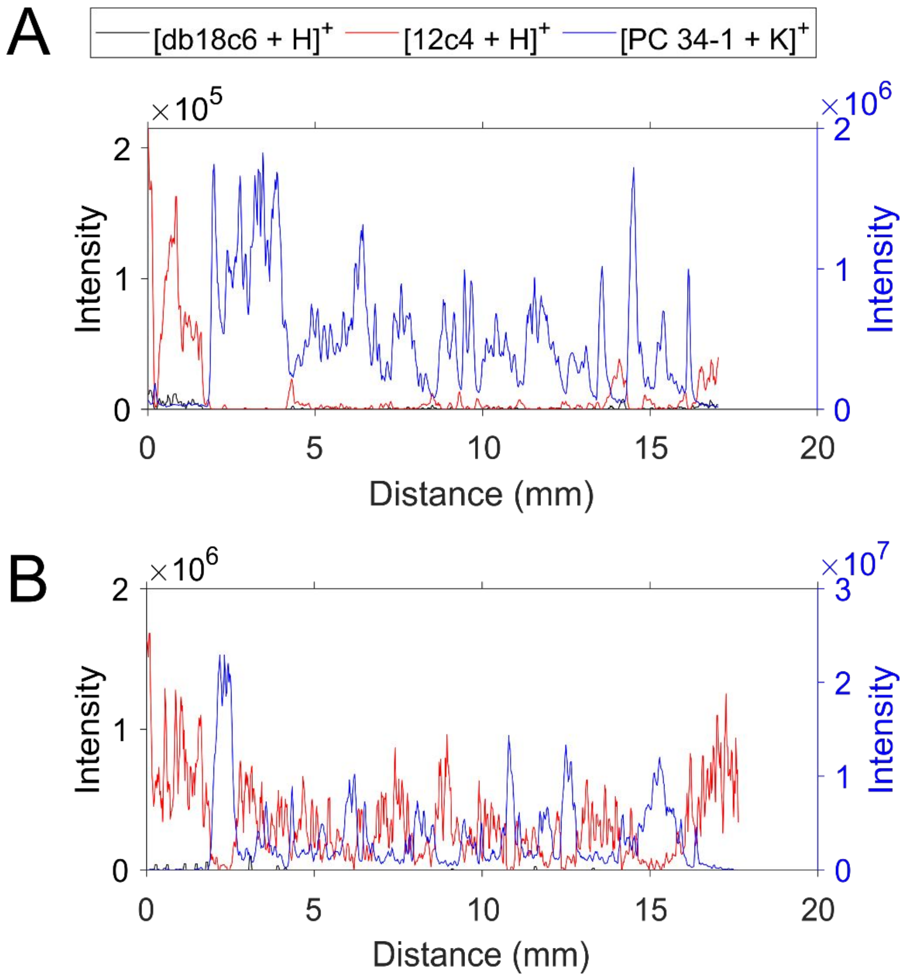

Figure S2. Line scan acquired over the sodium-spiked mimetic tissue model with nano-DESI MS where the solvent was A) MeOH:H_2O 9:1 v/v and B) MeOH:H_2O 9:1 v/v spiked with 0.1 % formic acid. Traces for protonated adducts of db18c6 (black line) and 12c4 (red line) are shown with the corresponding intensities depicted on the left y-axis. Trace for endogenous potassiated PC 34:1 from the mimetic tissue model (blue) is also shown with the corresponding intensity on the right y-axis.

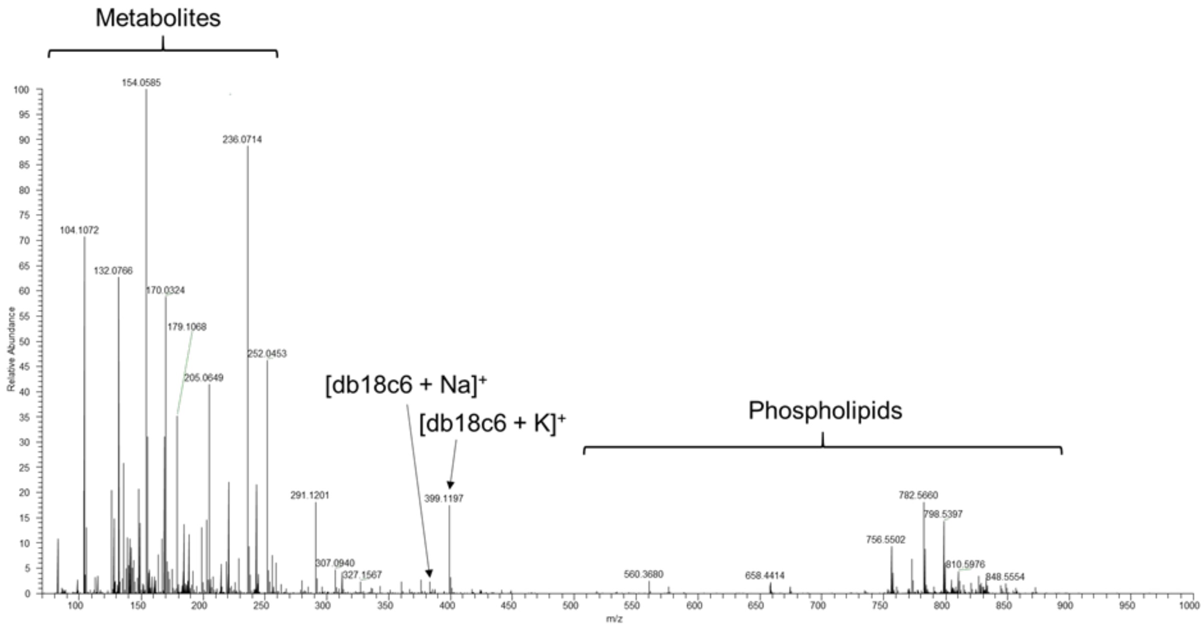

Figure S3. Mass spectrum obtained during db18c6 doped nano-DESI MSI of MCAO mouse brain tissue section representing one scan event.

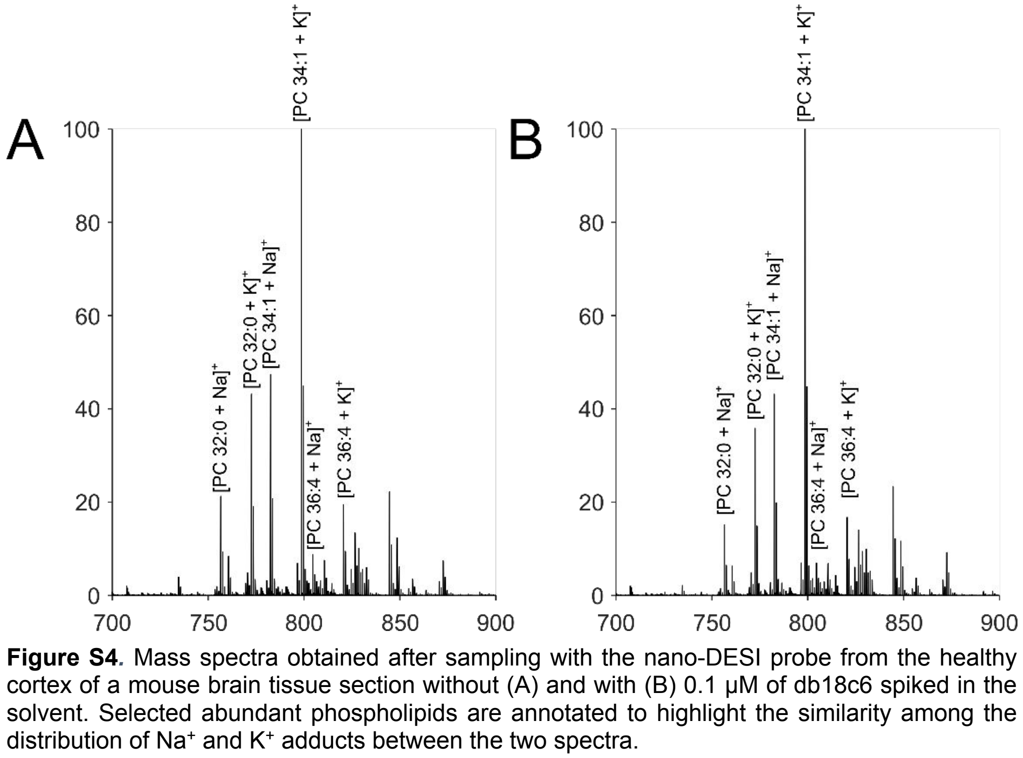

Figure S4. Mass spectra obtained after sampling with the nano-DESI probe from the healthy cortex of a mouse brain tissue section without (A) and with (B) 0.1 µM of db18c6 spiked in the solvent. Selected abundant phospholipids are annotated to highlight the similarity among the distribution of Na^+ and K^+ adducts between the two spectra.

Table S2. Binding affinities for Na^+ and K^+ for the various crown ethers used. All values obtained from: Izatt, R. M.; Pawlak, K.; Bradshaw, J. S.; Bruening, R. L. *Chem. Rev.* **1991**, *91* (8), 1721–2085

Crown ether	$\log(Kb_{Na^+})$	$\log(Kb_{K^+})$
12-crown-4	2.05	1.73
benzo-15-crown-5	3.24	3.43
15-crown-5	2.94	2.96
dibenzo-18-crown-6	4.37	5

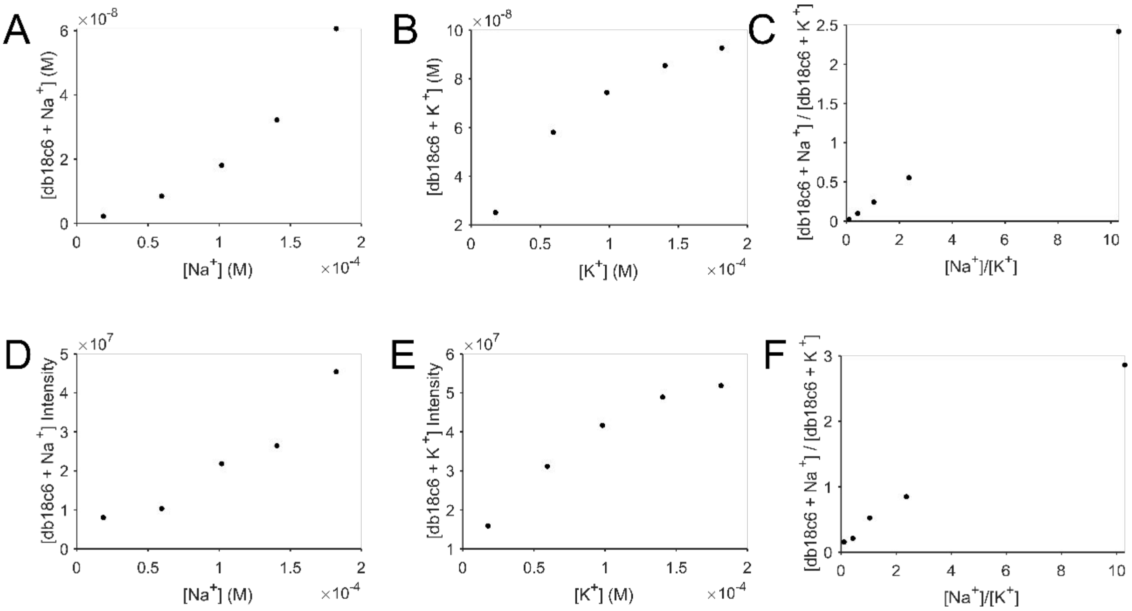

Figure S5. Top row: theoretical equilibrium concentrations of $[db18c6 + Na]^+$ vs $[Na^+]$ (A), $[db18c6 + K]^+$ vs $[K^+]$ (B) and their corresponding ratios (C) obtained at total salt concentration of 200 μM and various proportions of Na and K (10 % Na – 90 % K, 30 % Na – 70 % K, 50 % Na – 50 % K, 70 % Na – 30 % K and 90 % Na – 10 % K). Equilibrium concentrations of the complexes were obtained by solving simultaneous equilibrium equations (see section "Calculation of theoretical equilibrium concentrations for crown ether adducts with Na^+ and K^+" for details) in a system where both Na^+ and K^+ are present simultaneously and the crown ether concentration was 0.1 μM. Bottom row: Experimental data (mass spectrometric intensities) of $[db18c6 + Na]^+$ vs $[Na^+]$ (D), $[db18c6 + K]^+$ vs $[K^+]$ (E) and their corresponding ratios (F) obtained at total salt concentration of 200 μM and various proportions of Na^+ and K^+ (10 % Na – 90 % K, 30 % Na – 70 % K, 50 % Na – 50 % K, 70 % Na – 30 % K and 90 % Na – 10 % K). All solutions were analyzed with direct infusion ESI-MS and a constant crown ether concentration of 0.1 μM.

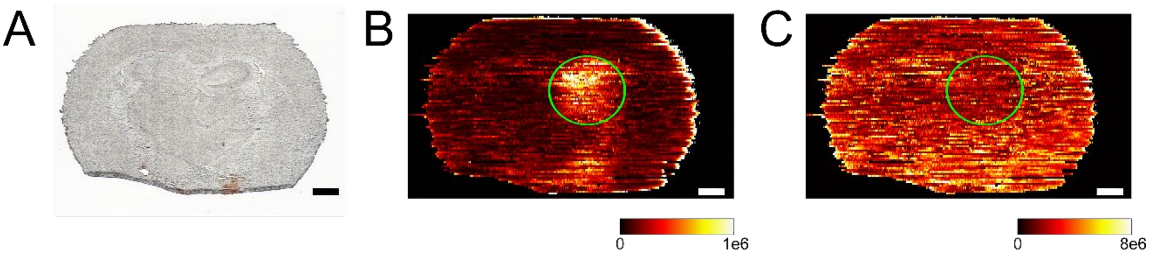

Figure S6. A) Optical image of MCAO mouse brain tissue section. B) Ion image of $[db18c6 + Na]^+$. C) Ion image of $[db18c6 + K]^+$. Scaling has been adjusted for visualization of less intense features. Only tissue related pixels are shown, as described in the main text. Green circles indicate the damaged area. Scale bars show 1 mm.

Table S3. Concentrations of Na^+ and K^+ for the various total salt conditions tested and for the different proportions of Na^+ and K^+ in each solution analyzed with pneumatically assisted nano-DESI.

	**Na^+ and K^+ concentration (μM) in each solution**									
	**10 % Na^+ 90 % K^+**		**30 % Na^+ 70 % K^+**		**50 % Na^+ 50 % K^+**		**70 % Na^+ 30 % K^+**		**90 % Na^+ 10 % K^+**	
**Total salt concentration (μM)**	Na^+	K^+	Na^+	K^+	Na^+	K^+	Na^+	K^+	Na^+	K^+
300	30	270	90	210	150	150	210	90	270	30
500	50	450	150	350	250	250	350	150	450	50
700	70	630	210	490	350	350	490	210	630	70
900	90	810	270	630	450	450	630	270	810	90
1100	110	990	330	770	550	550	770	330	990	110

Table S4. Predicted and experimentally determined adduct intensity ratios and % bias of the predictions for unknown adduct intensity ratios using the linear regression line experimentally determined by analyzing neat solutions using direct infusion ESI-MS in Figure S8.

**Predicted [Na^+]/[K^+]**	**Experimentally determined [Na^+]/[K^+] (± one standard deviation) (n=3)**	**% Bias**
0.24	0.25 (± 0.08)	4.2
0.71	0.65 (± 0.01)	-8.5
4.43	5.02 (± 0.58)	13.3

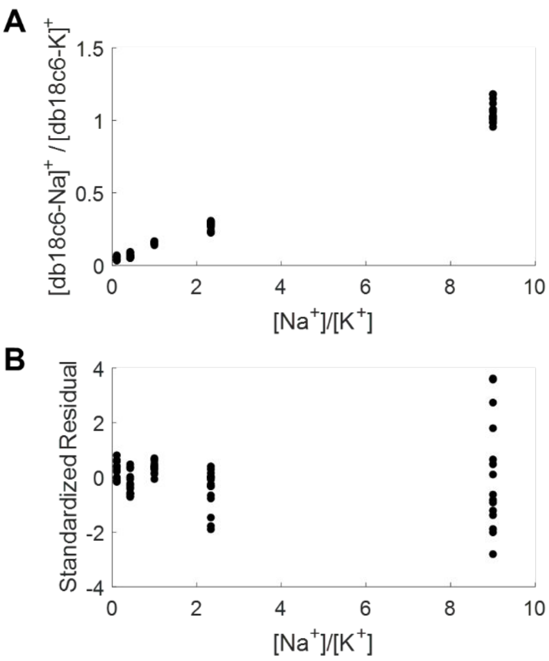

Figure S7. A) Na^+/K^+ adduct intensity ratio of db18c6 versus $[Na^+]/[K^+]$ at various total salt concentrations (see main text for details). B) Standardized residuals versus $[Na^+]/[K^+]$ obtained after ordinary least squares regression on the data shown in A.

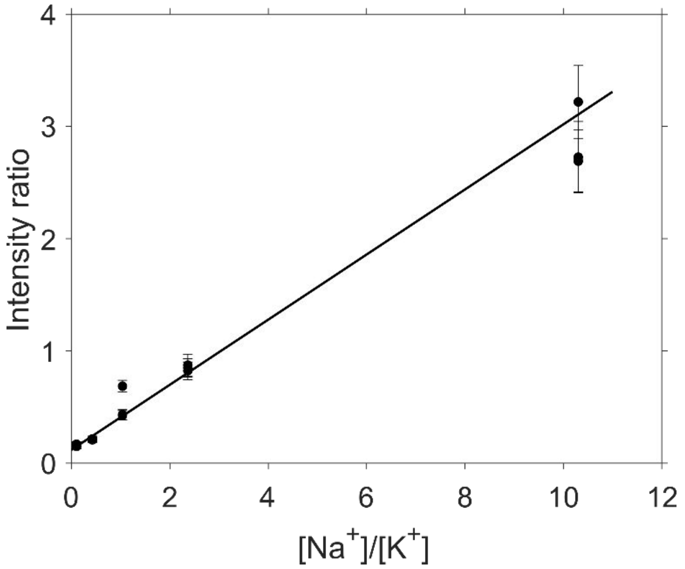

Figure S8. Linear regression of Na^+/K^+ db18c6 adduct intensity ratio experimentally obtained with direct infusion ESI-MS to predict the adduct intensity ratios of unknown solutions in Table S4. Total salt of neat solutions with various $[Na^+]/[K^+]$ (0.10, 0.42, 1.04, 2.36 and 10.3) at total salt concentration 200 μM dissolved in methanol. All solutions contained 0.1 μM of db18c6 and were analyzed in triplicates. Fitted line was obtained with weighted least squares with $1/\sigma^2$ was used as weighting. The equation of the line is $y = 0.29x + 0.12$.

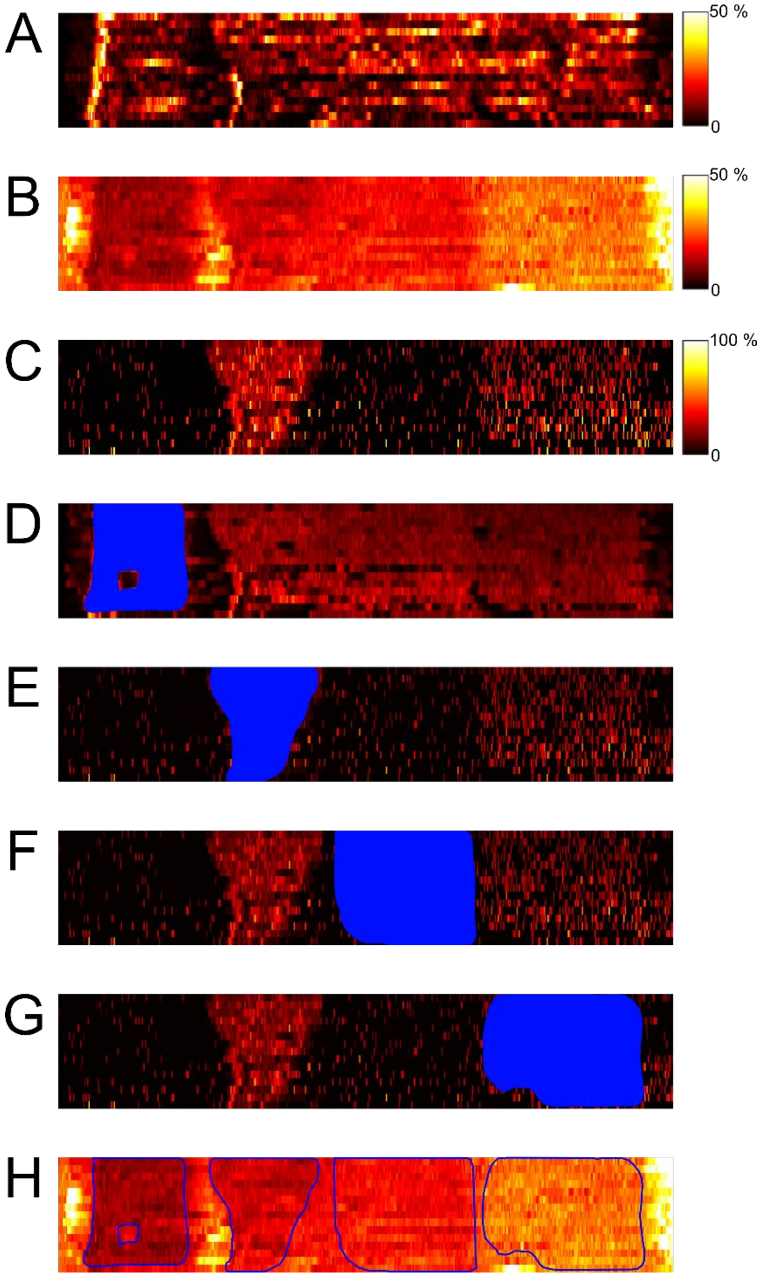

Figure S9. A) Ion image of endogenous [LPC 16:0 + Na]$^+$ normalized to [LPC 19:0 + Na]$^+$ in the mimetic tissue model spiked with NaCl. B) Na^+/K^+ adduct intensity ratio image of db18c6 C) Composite ion image of [enalapril + K]$^+$ and [GABA-^{15}N + Na]$^+$ spiked in the second and fourth layer of the mimetic tissue model, respectively. D) ROI selection of the first layer (0 μmol Na^+ g^{-1}) based on the distribution of endogenous [creatine + K]$^+$. E) ROI selection of the second layer (20 μmol Na^+ g^{-1}) based on the image shown in C. F) ROI selection of the third layer (50 μmol Na^+ g^{-1}) based on the image shown in C. G) ROI selection of the fourth layer (90 μmol Na^+ g^{-1}) based on the image shown in C. H) Na^+/K^+ adduct intensity ratio image of db18c6 with all ROI selections depicted in panels D-G combined.

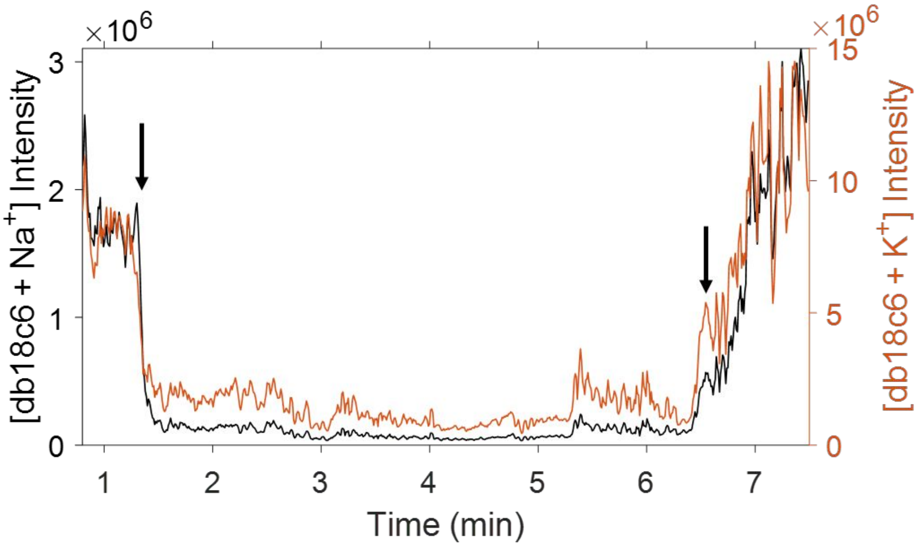

Figure S10. Mass spectrometric intensities of [db18c6 + Na]$^+$ (black line, left axis) and [db18c6 + K]$^+$ (orange line, right axis) when conducting surface sampling from rat brain tissue section. The area between the arrows indicates the tissue surface while outside the arrows the probe was sampling from the glass slide surface. Data were smoothed with a moving average (n = 3).

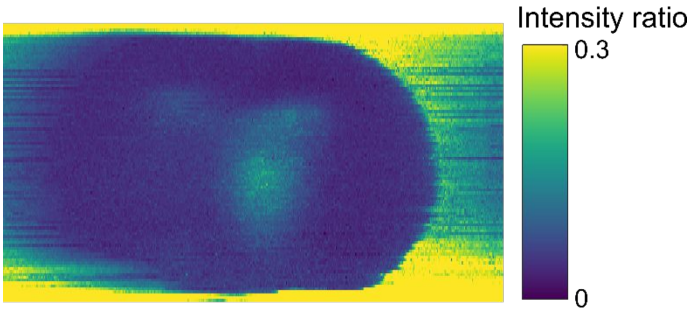

Figure S11. Na^+/K^+ adduct intensity ratio image of db18c6 obtained after nano-DESI MSI of MCAO mouse brain tissue section. The image shown here is unprocessed, i.e. all pixels are shown (both glass and tissue) compared to the image shown in Figure 4B where only tissue pixels are shown. The data acquisition was done by sampling from right to left. The intensity ratio is not constant throughout the glass slide since the high amount of K^+ extracted from the tissue is not immediately rinsed away and is carried over to the next acquired line.

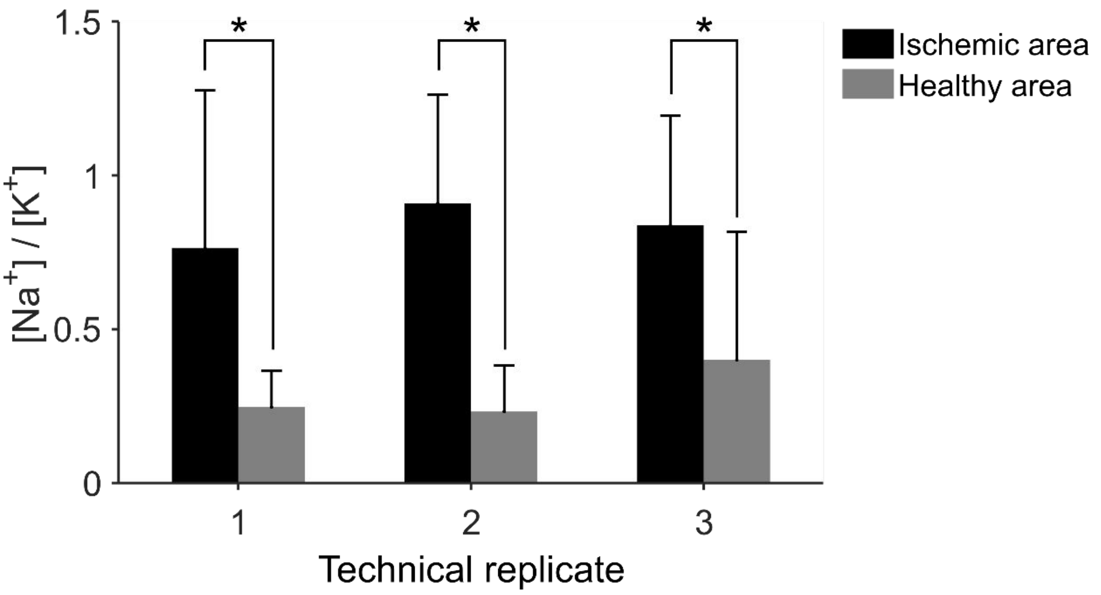

Figure S12. Average $[Na^+]/[K^+]$ obtained through ROI analysis of three independently analyzed MCAO mouse brain tissue sections. Error bars represent one standard deviation for each average (pixel-to-pixel variation). Statistical significance (* $p < 0.05$) between the healthy and ischemic area of each replicate was assessed using a two-tailed t-test.

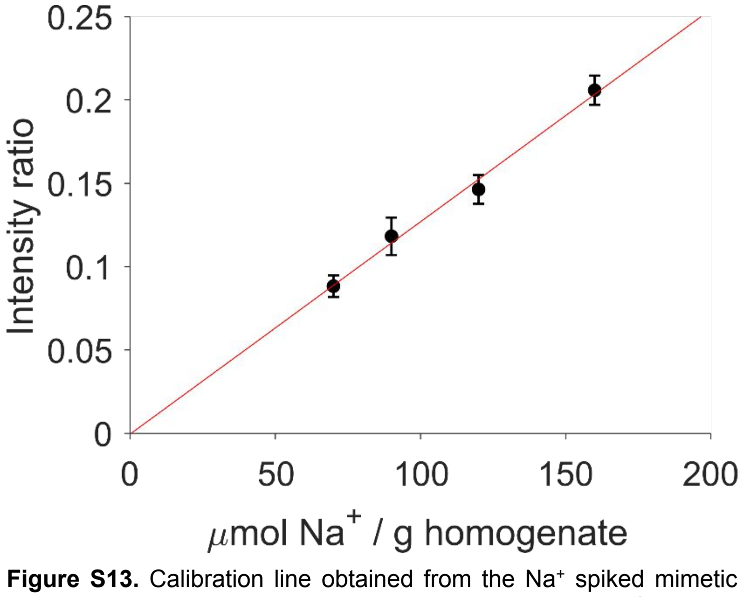

Figure S13. Calibration line obtained from the Na^+ spiked mimetic tissue model showing the Na^+/K^+ adduct intensity ratio of db18c6. Each x-axis point represents the Na^+ concentration in each layer. The concentration in the non-spiked layer (first point) was determined as described in the main text, through extrapolation from the standard curve of Figure 3C. Each x-axis point has been shifted by the concentration of the non-spiked homogenate (70 μmol Na^+ g^{-1}). Red line indicates linear fit through the data points with equation $y = 0.00127x - 0.000346$. Error bars show one standard deviation for each average intensity ratio.
